# Detecting plasmid-mediated dissemination of *bla*_KPC-3_ and *bla*_OXA-48-like_ genes in Enterobacterales across Finnish healthcare organizations using hybrid genome assembly

**DOI:** 10.3389/fmicb.2025.1567913

**Published:** 2025-06-17

**Authors:** Meeri Piispa, Anni Vainio, Jani Halkilahti, Outi Lyytikäinen, Kati Räisänen

**Affiliations:** ^1^Department of Microbiology, Faculty of Agriculture and Forestry, University of Helsinki, Helsinki, Finland; ^2^Department of Public Health, Microbiology Unit, Finnish Institute for Health and Welfare (THL), Helsinki, Finland

**Keywords:** hybrid assembly, horizontal plasmid-mediated gene transfer, outbreak, molecular epidemiology, plasmid, CPE, whole genome sequencing

## Abstract

The spread of carbapenemase-producing Enterobacterales (CPE) is a global concern. While the majority of the CPE outbreaks are due to clonal spread, recent findings highlight the transmission of carbapenemase gene-carrying plasmids across various bacterial species, exacerbated by extensive antibiotic use in hospitals. This study aimed to identify plasmid-mediated horizontal transfer of carbapenemase genes among Enterobacterales isolated from patient samples and hospital environment samples in three healthcare organizations in Finland. Using a hybrid assembly of short and long reads, we could complete the genome assembly and compare the plasmids harboring the *bla*_KPC-3_ and *bla*_OXA-48-like_ genes. Our findings reveal indications of interspecies and intraspecies plasmid-mediated gene transfer of *bla*_KPC-3_ and *bla*_OXA-48-like_, emphasizing the role of horizontal gene transfer (HGT) in outbreaks. The study underscores the need for comprehensive infection control and surveillance beyond specific species to prevent the spread of antimicrobial resistance genes. These results suggest that expanding outbreak investigations to an interspecies level could be beneficial.

## Introduction

Carbapenems are considered last-line drugs for the treatment of infections caused by multidrug-resistant Enterobacterales (Van Duin and Doi, 2017). The continuous rise in carbapenem resistance, resulting from the acquisition of carbapenemase genes, is a global concern. Infections caused by carbapenemase-producing Enterobacterales (CPE) are commonly associated with healthcare settings, where the hospital environment often serves as a reservoir for the spread of these bacteria. In outbreak investigations, the primary focus has usually been on tracking the clonal spread of a single pathogen. However, it is essential to consider that carbapenem resistance genes are predominantly located in mobile genetic elements (MGEs), such as integrons, insertion sequences, transposons, and plasmids (Kopotsa et al., 2019). It has been estimated that up to half of the CPE transmissions could occur through plasmid-mediated mechanisms (Marimuthu et al., 2022). The ability of plasmids to harbor multiple antibiotic resistance genes (ARGs) and facilitate their transfer between the same and different bacterial species makes them highly significant in the molecular epidemiology of CPE (Kopotsa et al., 2019). Horizontal gene transfer (HGT) of plasmids via conjugation occurs through physical contact between bacteria. This process involves plasmids carrying mobility (MOB) genes for DNA processing and a mating pair formation (MPF) complex, a type 4 secretion system (T4SS), to form the mating channel (Smillie et al., 2010; Coluzzi et al., 2022). Plasmids can be classified as conjugative (self-transmissible), mobilizable (relying on another element’s MPF genes), or non-mobilizable. Conjugation begins with a relaxase enzyme nicking the plasmid DNA at the origin of transfer (oriT), thereby initiating rolling-circle replication in the donor cell (Coluzzi et al., 2022). The resulting single-stranded DNA is then transferred to the recipient cell via the T4SS, where it is circularized and replicated to restore its double-stranded form.

While the majority of the *Klebsiella pneumoniae* carbapenemase (KPC)-producing *Klebsiella pneumoniae* outbreaks reported to date are due to clonal spread (Marí-Almirall et al., 2021; Pournaras et al., 2009; van Beek et al., 2019), recent findings suggest an emerging concern regarding the transmission of KPC gene-carrying plasmids (Adler et al., 2016), facilitating their dissemination across different bacterial species and genera (Schweizer et al., 2019). Plasmid-mediated horizontal transfer of resistance genes, often exacerbated by the extensive use of antibiotics in hospitals, may play a significant role in the regional and supra-regional spread of carbapenem resistance in healthcare settings (Li et al., 2018; Marí-Almirall et al., 2021; Schweizer et al., 2019).

To date, the number of CPE cases has been relatively low in Finland, ranging from 50 to 120 cases annually (Finnish Institute for Health and Welfair, 2023). From 2017 to 2022, on average, one-third of the annual CPE strains were associated with possible local transmission, indicating that they were genetically closely related. The most common types of carbapenemases detected were *bla*_KPC_, *bla*_NDM_, and *bla*_OXA-48-like_.

This study aimed to identify the interspecies and intraspecies plasmid-mediated horizontal transfer of carbapenemase genes among Enterobacterales isolated from patient samples and hospital environment samples in three healthcare organizations in Finland. The location of the *bla*_KPC-3_ and *bla*_OXA-48-like_ genes was investigated in the bacterial genome and plasmid components for mobility prediction. Knowing the transmission routes of the gene will provide valuable information for tackling CPE outbreaks and epidemiological surveillance.

## Materials and methods

According to the Communicable Diseases Act (1227/2016) and the national guidelines for controlling multidrug-resistant microbes (Kolho et al., 2020), all Finnish clinical microbiology laboratories are required to notify the National Infectious Diseases Register of any human isolates of *Enterobacter cloacae, Escherichia coli*, and *K. pneumoniae* that exhibit reduced susceptibility to carbapenems. These bacterial strains must also be submitted to the national CPE strain collection at the Finnish Institute for Health and Welfare (THL) (Räisänen et al., 2020). In addition, other CPE species and environmental isolates obtained as a part of the outbreak investigations are sent for further characterization to THL. Species identification and antimicrobial susceptibility tests were performed in clinical microbiology laboratories, along with confirmation of carbapenemase genes for isolates with reduced susceptibility, as previously described (Räisänen et al., 2020). For short-read whole genome sequencing (WGS), 1 ng of purified DNA was used, and the library was prepared using the Nextera XT DNA Sample Preparation Kit (Illumina, SD, USA). The paired-end short reads (2× 150-bp) were sequenced using the Illumina MiSeq instrument (Illumina, SD, USA). The short-read sequences were processed and analyzed using Trimmomatic (version 0.33), fastQC (version 0.11.6), SRST2 (version 0.2.0), and SeqSphere+ (Ridom GmbH, Münster, Germany), as previously described (van Beek et al., 2019).

When selecting CPE strains for this study, patient, time, and hospital environment were noted and grouped accordingly (Table 1). KP_4 and EC_1 strains were obtained from the same patient on the same collection date, both carrying the *bla*_OXA-48-like_ gene (group 1). The other eight strains were obtained within a close timeframe in the same or close location to each other, all carrying the *bla*_KPC-3_ gene (groups 2A and 2B). Locations A and C were related to an outbreak caused by *K. pneumoniae* ST512 (van Beek et al., 2019) and two other *K. pneumoniae* strains (KP_1 & KP_2). *Citrobacter freundii* strain (CF_1) from a cluster *C. freundii* ST116 (Räisänen et al., 2021) was obtained from the same patient as the KP_2 strain (group 2B). The environmental strains of *Klebsiella oxytoca* (KO_1), *Citrobacter braakii* (CB_1), and *Enterobacter agglomerans* (EA_1) were obtained from the same hospital ward.

**Table 1:** Characteristics of the study strains.

ID	Group	Species	Sequence type	Carbapenemase gene	Collection date	Sources	Healthcare organization
KP_4	1	*K. pneumoniae*	ST15	*bla*_OXA-48-like_	04/2022	Human^1^	B
EC_1	1	*E. coli*	ST1170	*bla*_OXA-48-like_	04/2022	Human^1^	B
KP_1	2A	*K. pneumoniae*	ST512	*bla*_KPC-3_	10/2019	Hospital environment (toilet)	A
KP_3	2A	*K. pneumoniae*	ST307	*bla*_KPC-3_	03/2020	Human	A
CF_2	2A	*C. freundii*	ST125	*bla*_KPC-3_	08/2020	Hospital environment (floor drain)	C
KO_1	2A	*K. oxytoca*	ST21	*bla*_KPC-3_	08/2019	Hospital environment (toilet)*	C
CB_1	2A	*C. braakii*	NT	*bla*_KPC-3_	10/2019	Hospital environment (toilet)*	C
EA_1	2A	*E. agglomerans*	NT	*bla*_KPC-3_	12/2019	Hospital environment (floor drain)*	C
KP_2	2B	*K. pneumoniae*	ST512	*bla*_KPC-3_	01/2020	Human^2^	A
CF_1	2B	*C. freundii*	ST116	*bla*_KPC-3_	01/2020	Human^2^	A

NT, non-typable; KP, *Klebsiella pneumoniae*; CF, *Citrobacter freundii*; KO, *Klebsiella oxytoca*; CB, *Citrobacter braakii*; EA, *Enterobacter agglomerans*; EC, *Escherichia coli*.

1 Isolated from the same patient.

2 Isolated from the same patient.

*Isolated from the same hospital ward.

### Whole genome sequencing with Oxford Nanopore

The long-read WGS was performed using the MinION Mk1B device by the R9.4.1 flow cells (FLO-MIN106) (Oxford Nanopore Technologies, UK). The bacterial isolates were cultured from frozen stocks (−70°C) on Mueller-Hinton II agar plates (containing 2 g of beef extract, 17.5 g of acid hydrolysate of casein, 1.5 g of starch, and 17 g of agar) overnight at 37°C.

The library was prepared using the Rapid Barcoding Kit 96 (SQK-RBK110.96) (Oxford Nanopore Technologies, UK) according to the manufacturer’s protocol, except the eluate was incubated in the rotator mixer (1,200 rpm) for 10 min at 56°C. The total run time was 72 h.

Basecalling was performed using the Guppy basecaller (v6.3.8) in super high-accuracy mode in real time. The data were processed using the MinKNOW (v22.10.7) software with the following utility programs: Bream (v7.3.2) and Configuration (v5.3.7).

### Data analysis

For the data analysis, a FullForcePlasmidAssembler (FFPA) pipeline was used,^1^ which includes Trimmomatic (v0.39), QCAT (v1.1.0), UniCycler (v0.4.7), and NanoPlot (v1.30.1). The FFPA uses Trimmomatic and QCAT for trimming long and short reads. UniCycler (using eight threads) was used to maintain the hybrid assembly of short and long reads (Wick et al., 2017) using the *de novo* assembler SPAdes (v3.13.1) for the short reads (Bankevich et al., 2012), followed by alignment of the long reads to the graph. The quality and statistics of the MinION run were confirmed using NanoPlot (De Coster et al., 2018).

A software platform, Geneious Prime (v2022.2)^2^, was used to annotate and assemble the contigs found from the hybrid assembly FASTA data. The target genes (*bla*_KPC-3_ and *bla*_OXA-48-like_) were annotated against the contigs. The accession numbers of the genes were obtained from the ResFinder (v.4.1) (Bortolaia et al., 2020) (*bla*_KPC-3_: HM769262 and *bla*_OXA-48-like_: AY236073), and the corresponding gene sequences were obtained from the National Center for Biotechnology Information (NCBI) GenBank^3^. The genes were annotated against the contigs, and the target contigs were aligned using the MAFFT alignment -tool (v7.490) (Katoh and Standley, 2013). A distance matrix and a heatmap, as well as a similarity dendrogram, were built from the aligned contigs. The similarity dendrogram was built using Geneious Tree Builder with the Jukes–Cantor distance model and the neighbor-joining building method. Each target contig was compared to the NCBI^4^ database using the BLAST Megablast online tool.

The draft assembly of the plasmids was analyzed and characterized by the software tool MOB-suite using default parameters (Robertson and Nash, 2018). The MOB-suite includes a set of modular tools for reconstruction and typing. In the analysis, we used MOB-typer for conjugative transferability predictions. For predicting putative conjugative transferability, the MOB-typer identifies different DNA markers needed for the transfer, including an origin of transfer (oriT), a DNA relaxase, a type IV coupling protein (T4CP), and the type IV secretion system (T4SS) (Robertson and Nash, 2018). Plasmids possessing both a relaxase and a mate-pair formation marker were categorized as “conjugative,” plasmids that had either relaxase or oriT, but lacked the mate-pair formation marker, were classified as “mobilizable,” and plasmids that lacked both relaxase and oriT were considered “non-mobilizable.” Each plasmid replicon (rep) type was confirmed additionally using PlasmidFinder, with >95% identity and >85% coverage^5^ (Camacho et al., 2009; Carattoli et al., 2014).

### Ethical statement

The isolates were part of the CPE surveillance or outbreak investigations based on the Communicable Disease Act (1227/2016); therefore, patients were not contacted, and ethical permission was not needed.

## Results

The *bla*_OXA-48-like_ gene was detected in two strains (group 1), and the *bla*_KPC-3_ gene was detected in eight strains (groups 2A and 2B) (Table 1). In Geneious Prime, hybrid assembly data were divided into 3–14 contigs, and the size of the contigs where the target gene (*bla*_KPC-3_ or *bla*_OXA-48-like_) was found varied between 59,633 bp and 117,396 bp (Table 2). Each of the contigs with the carbapenemase gene was determined as a plasmid sequence with the grade (E-value 0) varying from 72.3 to 100%.

**Table 2:** Result of the BLAST search for the target contigs containing the target gene (*bla*_KPC-3_ or *bla*_OXA-48-like_).

ID	Carbapenemase genes	Number of contigs	Length of the target contigs (bp)	Grade % (*E*-value 0)	Type of element
KP_1	*bla*_KPC-3_	6	117,396	72.5	Plasmid
KP_2	*bla*_KPC-3_	6	117,058	72.5	plasmid
KP_3	*bla*_KPC-3_	5	114,528	85.6	Plasmid
KP_4	*bla*_OXA-48-like_	4	63,589	100	Plasmid
CF_1	*bla*_KPC-3_	4	112,552	72.3	Plasmid
CF_2	*bla*_KPC-3_	13	109,450	86.4	Plasmid
KO_1	*bla*_KPC-3_	14	59,633	95.6	Plasmid
CB_1	*bla*_KPC-3_	13	66,259	91	Plasmid
EA_1	*bla*_KPC-3_	3	116,907	81.9	Plasmid
EC_1	*bla*_OXA-48-like_	5	63,589	100	Plasmid

MOB-typer analysis revealed that 6 out of 10 (KP_3, KP_4, CF_1, CF_2, EA_1, and EC_1) plasmids were putatively conjugative and the remaining were putatively non-mobilizable (KP_1, KP_2, KO_1, and CB_1) (Table 3). The *bla*_OXA-48-like_ gene was located in putatively conjugative plasmids in both strains of group 1. In groups 2A and 2B, only the plasmids of the strains KP_3, CF_1, CF_2, and EA_1 containing the gene *bla*_KPC-3_ were classified as conjugative.

**Table 3:** Predicted mobility and rep type(s) of the plasmids containing the target gene (*bla*_KPC-3_ or *bla*_OXA-48-like_).

ID	Group	Predicted mobility	Rep type(s) (MOB-suite)	Rep type(s) (PlasmidFinder)
KP_4	1	Conjugative	IncL/M	IncL
EC_1	1	Conjugative	IncL/M	IncL
KP_1	2A	Non-mobilizable	IncFIB, IncFII, and rep_cluster_2183	IncFIB(pQil)
KP_3	2A	Conjugative	IncFIB, IncFII, and rep_cluster_2183	IncFIB(pQil) and IncFII(K)
CF_2	2A	Conjugative	IncFIC and IncFII	IncFII(SARC14)
KO_1	2A	Non-mobilizable	IncFIB, IncFII, and rep_cluster_2183	IncFII(K)
CB_1	2A	Non-mobilizable	IncFIB, IncFII, and rep_cluster_2183	IncFII(K)
EA_1	2A	Conjugative	IncFIB, IncFII, and rep_cluster_2183	IncFII(K)
KP_2	2B	Non-mobilizable	IncFIB, IncFII, and rep_cluster_2183	IncFIB(pQil)
CF_1	2B	Conjugative	IncFIB, IncFII, and rep_cluster_2183	IncFIB(pQil)

The last two columns are results from MOB-suite and PlasmidFinder, respectively.

**Figure 1:**
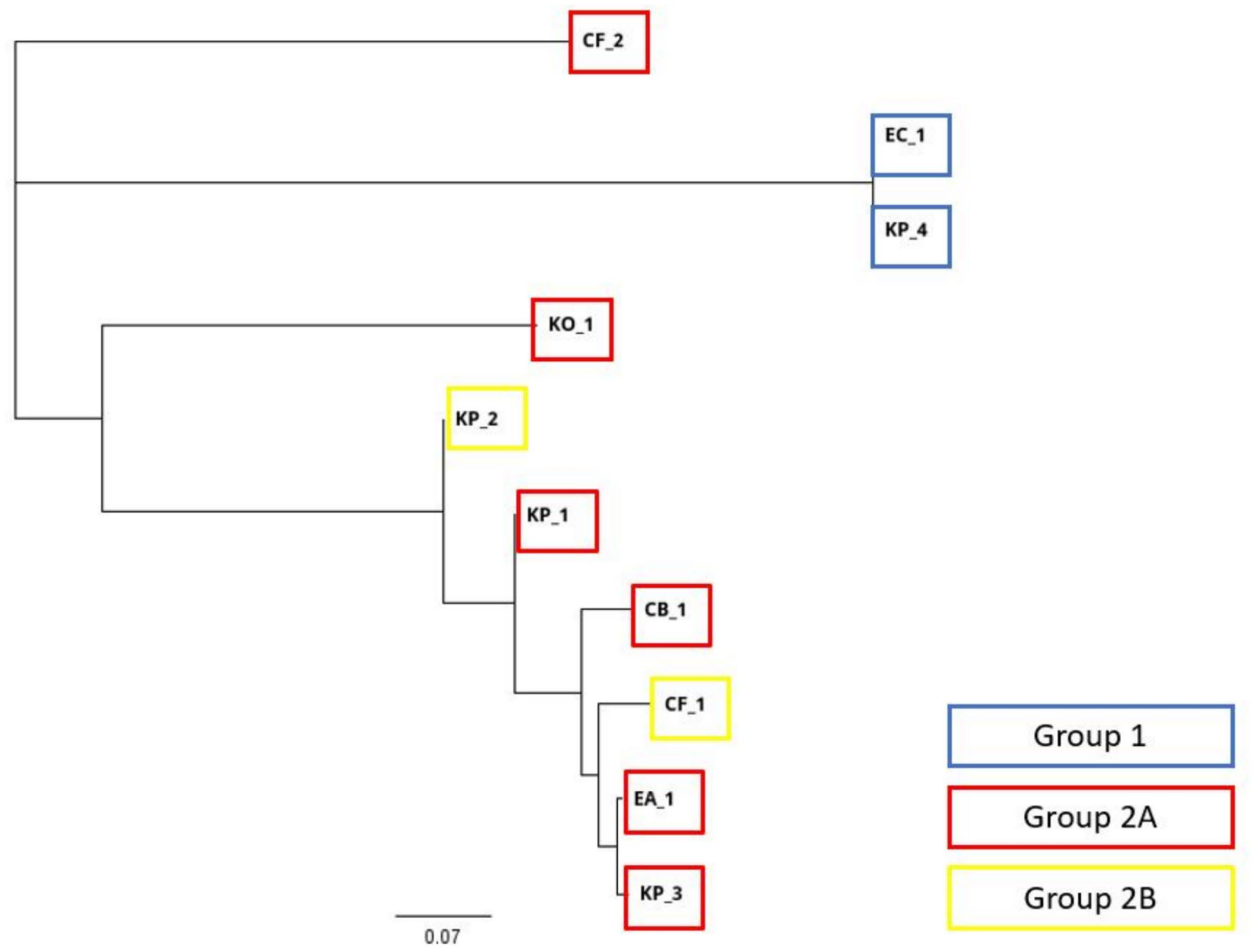
A dendrogram representing similarity of the contigs with carbapenemase gene (*bla*_KPC-3_ or *bla*_OXA-48-like_). The blue square marks the strains from group 1, red marks the strains from group 2A, and yellow marks the strains from group 2B.

The similarity dendrogram and distance matrix were generated by aligning the plasmid sequences that harbor the target genes (*bla*_KPC-3_ or *bla*_OXA-48-like_). Analysis of the similarities unveiled the clustering of plasmids into three distinct clades (Figure 1). The plasmids of EC_1 and KP_4 belonged to group 1 and created its own clade. The plasmids of KP_1, KP_2, KP_3, CF_1, KO_1, CB_1, and EA_1 belonging to group 2A and the plasmids belonging to group 2B were all found in the same clade. One plasmid from group 2A, namely CF_2, was found in its own clade apart from the rest of the plasmids.

The distance matrix and the heat map include the sequences of the contigs containing the target genes (*bla*_KPC-3_ or *bla*_OXA-48-like_) (Table 4). In this matrix, a higher value indicates a greater similarity in plasmid sequences. The total range of sequence similarity varied from 11 to 100%. In the distance matrix, the similarity between the plasmids in group 1 (with KP_4 and EC_1) was 100%. Among the largest clade—containing group 2A excluding CF_2 and group 2—the similarity ranged from 17 to 97%. In group 2A, the greatest similarity was observed between the putatively conjugative plasmids of KP_3 and EA_1, with a similarity of 97%. However, the putatively conjugative plasmid from CF_2 did not demonstrate significant similarities with the plasmids of any other strains. The plasmids of CF_1 and KP_2 from group 2B showed 81% similarity, although only CF_1 was characterized as putatively conjugative. Finally, among groups 2A and 2B, the putatively conjugative plasmid of CF_1 demonstrated a relatively close similarity (87%) with the putatively conjugative plasmids of KP_3 and EA_1.

**Table 4:** Distance matrix and the heat map of the sequences of the contigs containing the target genes (*bla*_KPC-3_ or *bla*_OXA-48-like_).

Group		1		2A						2B	
	Isolate	**EC_1**	**KP_4**	CB_1	**CF_2**	**EA_1**	**KP_3**	KP_1	KO_1	KP_2	**CF_1**
1	**EC_1**	**x**	**100**	**13**	**22**	**20**	**20**	**21**	**25**	**21**	**20**
	**KP_4**	**100**	**x**	**13**	**22**	**20**	**20**	**21**	**25**	**21**	**20**
2A	CB_1	**13**	**13**	x	**11**	**53**	**54**	49	17	47	**52**
	**CF_2**	**22**	**22**	**11**	**x**	**25**	**24**	**29**	**28**	**29**	**24**
	**EA_1**	**20**	**20**	**53**	**25**	**x**	**97**	**89**	**29**	**86**	**87**
	**KP_3**	**20**	**20**	**54**	**24**	**97**	**x**	**88**	**29**	**85**	**87**
	KP_1	**21**	**21**	50	**30**	**89**	**88**	x	38	93	**84**
	KO_1	**25**	**25**	17	**28**	**29**	**29**	38	x	41	**29**
2B	KP_2	**21**	**21**	47	**29**	**86**	**85**	93	41	x	**81**
	**CF_1**	**20**	**20**	**52**	**24**	**87**	**87**	**84**	**29**	**81**	**x**

□, 0–19%; 
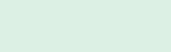
, 20–39%;
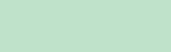
, 40–59%;
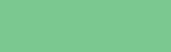
, 60–79;
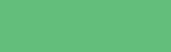
, 80–100%.

Conjugative plasmid sequences are in bold.

## Discussion

With the hybrid assembly, we could overcome the challenge stated in the article by Zou et al. (2022), where short-read sequencing often resulted in fragmented genomes, thereby complicating the classification of chromosomal and plasmid sequences. By using the hybrid assembly, we were able to create a comprehensive assembly of the plasmids and map the target genes (*bla*_KPC-3_ or *bla*_OXA-48-like_). Based on the MOB-typer analysis, 6 out of 10 strains carried putatively conjugative plasmids (Table 3). By comparing the similarities between the plasmid sequences, we could predict the mobility between the strains and predict if interspecies and intraspecies plasmid-mediated HGT has occurred.

The strains KP_4 and EC_1 from group 1, isolated from the same patient, carried the *bla*_OXA48-like_ gene on identical, putatively conjugative plasmids classified as IncL/M type (Tables 2, 3). These plasmids, found in the same clade and with 100% similarity, strongly suggest interspecies plasmid-mediated HGT (Figure 1). This finding aligns with previous research by Hamprecht et al. (2019), which demonstrated that *bla*_OXA48_ dissemination primarily occurs via plasmid-mediated HGT rather than clonal expansion. In addition, MOB-typer and PlasmidFinder analyses confirmed the IncL/M and IncL rep types, respectively, which was consistent with the study by Poirel et al. (2012), further supporting the notion of a common origin for *bla*_OXA48-like_-carrying plasmids. These plasmids exhibit low fitness burden and high stability, enhancing HGT potential (Hamprecht et al., 2019).

In group 2A, only strains KP_3, CF_2, and EA_1 harbored putatively conjugative plasmids according to the MOB-typer. The plasmid sequences of KP_3 and EA_1 exhibited a high similarity (97%) (Tables 3, 4), and this high similarity strongly suggests plasmid-mediated HGT (Orlek et al., 2017; Schweizer et al., 2019). The presence of these strains in environmental and human samples supports the hypothesis that environmental contamination in hospitals contributes to the transmission of the *bla*_KPC-3_ gene among bacteria, as previously suggested by van Beek et al. (2019). In addition, the similarity in plasmid sequences and mobility between different species implies that interspecies plasmid-mediated gene transfer of the *bla*_KPC-3_ gene likely occurred between environmental and human isolates. Marí-Almirall et al. (2021) proposed a similar gene transmission dynamic for the *bla*_KPC-2_ gene in their investigation of the first hospital outbreak caused by KPC-producing Enterobacterales in Catalonia, reporting intraspecies and interspecies transmission associated with plasmid-mediated gene transfer. They observed the plasmid overcoming genetic rearrangements in non-*K. pneumoniae* isolates, which could explain the minor differences observed in plasmid sequences in our study.

The suspected plasmid-mediated HGT between the environmental strains KO_1, CB_1, and EA_1 from the same hospital ward is difficult to confirm, as only the plasmid found in EA_1 was classified as putatively conjugative. In addition, the plasmid sequence of KO_1 exhibited low similarity with CB_1 (17%) and EA_1 (29%). The similarity between CB_1 and EA_1 was higher at 53%. Thus, confirming any conclusions would require more investigation.

Group 2B strains, isolated from the same patient within a short interval, suggested potential interspecies HGT (Evans et al., 2020). However, the plasmid of strain CF_1 was classified as conjugative; however, that of strain KP_2 was classified as non-mobilizable (Table 3), and moreover, the similarity of the plasmids was 81% (Table 4). It is possible that plasmid-mediated HGT occurred between CF_1 and KP_2, but the plasmid may have evolved and lost its autonomous conjugative ability (Coluzzi et al., 2022). Although the plasmid size of KP_2 was larger than that of CF_1 (Table 2), the observed differences may not be due to a simple deletion; instead, recombination events could have occurred, leading to the loss of genes required for conjugation. Confirming this would require further investigation into the plasmid sequences. Among groups 2A and 2B, all plasmids belonged to groups IncFIB and IncFII. In addition, all except CF_2 also had rep_cluster_2183. Only KP_3, CF_2, EA_1, and CF_1 were classified as containing a putatively conjugative plasmid with the target gene. The plasmid sequence of the strain CF_1 shared great similarity (>80%) with the plasmid sequences of the strains KP_3, KP_2, and EA_1. The strains CF_1 and KP_3, obtained from human samples collected 2 months apart at healthcare organization A, strongly suggest the occurrence of interspecies plasmid-mediated HGT among patients within the same healthcare organization. This finding confirms the discovery by Li et al. (2018) that isolates originating from a single hospital have the ability to spread among various species of Enterobacteriaceae, indicating a wide dissemination of the plasmids within the hospital. The connection observed between strains CF_1 and EA_1 further supports the previously mentioned pattern of interspecies plasmid-mediated HGT between environmental and human samples. No specific bacterial species was found to be more prone to plasmid-mediated HGT.

The plasmids of the strains KP_2 and KP_1, which are related to the same cluster (van Beek et al., 2019), created their own subgroup with 93% similarity as expected, as they were identified to be involved in the clonal spread based on the cgMLST analysis (by using Illumina data only) and shared a common epidemiological link. Predicted mobility analysis demonstrated their non-mobilizable status, further validating clonal spread and likely ruling out plasmid-mediated HGT between the strains.

In this study, we showed a strong indication of interspecies plasmid-mediated gene transfer of antibiotic resistance genes *bla*_KPC-3_ and *bla*_OXA-48-like_. This study highlights the prevalence of HGT in outbreaks and that infection control and surveillance should not only concern a specific species. In such outbreaks, extensive detection and surveillance should be designated as a prevention of the spread of the AMR genes. The plasmid-mediated *bla*_KPC-2_ gene transfer in multispecies outbreaks has been reported worldwide, for example, in Germany (Schweizer et al., 2019) and China (Li et al., 2018). Based on our results, expanding the outbreak investigation to the interspecies level in Finland should be considered in the future.

Our study has several limitations. The prediction of plasmid-mediated HGT was based solely on observing the putative mobility and similarity of the plasmids. Performing a phylogenetic analysis of plasmids is challenging because they often lack conserved core genes, and sequence dissimilarity does not necessarily indicate a distant common origin (Orlek et al., 2017). Plasmids that are phylogenetically distant may share genetic content due to the insertion of similar mobile elements (Redondo-Salvo et al., 2020), while closely related plasmids can exhibit significant sequence differences after recombination with other genetic structures (Redondo-Salvo et al., 2020; Schweizer et al., 2019). Therefore, plasmid similarity might not be the best indicator of HGT. The use of MOB-typer for mobility analysis should be approached with caution and validated with another method in addition. Approximately half of the plasmids were identified as non-mobilizable, meaning that they lack relaxase and oriT, which are essential for the conjugation process. There is a possibility that these strains carry novel oriT systems in their plasmids that are not recognized. This could be explained by recent findings by Ares-Arroyo et al. (2024), which suggest that many oriTs are currently unrecognized. In addition, studies have found that plasmids with incomplete conjugation systems, lacking some essential genes, can utilize other mobile genetic elements (MGEs) such as bacteriophages or other plasmids to facilitate mobility (Ares-Arroyo et al., 2024; Coluzzi et al., 2022). To better understand this phenomenon, further investigation into other MGEs in the bacterium’s genome is required.

To confirm both mobility and HGT in future studies, it would be advisable to perform plasmid dissemination tests, including conjugation assays between isolates, and investigation of the MGEs. In addition, our sample size was relatively small, limiting the ability to make definitive conclusions. Further data are required to strengthen our findings.

## Conclusion

In this study, by using the hybrid assembly of short and long reads, we could successfully distinguish the bacterial genome into contigs to separate plasmid and chromosomal sequences, investigate whether the carbapenem resistance gene is in the plasmid or not, and predict whether the gene spreads horizontally between the strains. To the best of our knowledge, this is the first report where the plasmid-mediated spread of the AMR gene in Finland is investigated by using the hybrid assembly. Understanding the transmission route of the gene could yield valuable insights for addressing CPE outbreaks, as it has been assessed that 50% of them are disseminated via plasmids (Marimuthu et al., 2022). The threshold for indications of HGT is difficult to determine with such small sampling. To more precisely determine the exact threshold and other indications of the plasmid-mediated HGT, more research is required.

## Data Availability

The datasets presented in this study can be found in online repositories. The names of the repository/repositories and accession number(s) can be found at: https://www.ebi.ac.uk/ena, PRJEB84916.

## Footnotes

^1^https://github.com/MBHallgren/FullForcePlasmidAssembler#readme/ accessed 2023-2-8

^2^ https://www.geneious.com

^3^https://www.ncbi.nlm.nih.gov/nucleotide/ accessed 2023-8-18

^4^https://blast.ncbi.nlm.nih.gov/Blast.cgi/ accessed 2023-8-18

^5^http://cge.cbs.dtu.dk/services/PlasmidFinder/ accessed 2025-5-7

## References

[ref1] Adler A. Efrat K. Paikin S. Carmeli Y. (2016). Dissemination of the BlaKPC gene by clonal spread and horizontal gene transfer: comparative study of incidence and molecular mechanisms. J. Antimicrob. Chemother. 71, 2143–2146. doi: 10.1093/jac/dkw106 27073266

[ref2] Ares-Arroyo M. Nucci A. Rocha E. P. C. (2024). Identification of novel origins of transfer across bacterial plasmids. Available online at: https://www.biorxiv.org/content/10.1101/2024.01.30.577996v1. (Accessed November 13, 2024)

[ref9003] Bankevich A. Nurk S. Antipov D. Gurevich A. A. Dvorkin M. Kulikov A. S. (2012). “SPAdes: a new genome assembly algorithm and its applications to single-cell sequencing.” Journal of Computational Biology: A Journal of Computational Molecular Cell Biology, 19, 455–477. doi: 10.1089/cmb.2012.0021 22506599 PMC3342519

[ref9004] Bortolaia V. Kaas R. S. Ruppe E. Roberts M. C. Schwarz S. Cattoir V. (2020). “ResFinder 4.0 for predictions of phenotypes from genotypes.” The Journal of Antimicrobial Chemotherapy, 75, 3491–3500. doi: 10.1093/jac/dkaa345 32780112 PMC7662176

[ref4] Camacho C. Coulouris G. Avagyan V. Ma N. Papadopoulos J. Bealer K.. (2009). BLAST+: architecture and applications. BMC Bioinformatics 10:421. doi: 10.1186/1471-2105-10-421 20003500 PMC2803857

[ref5] Carattoli A. Zankari E. Garcia-Fernandez A. Voldby Larsen M. Lund O. Villa L.. (2014). Plasmidfinder and pMLST: in silico detection and typing of plasmids. Antimicrob. Agents Chemother. 58, 3895–3903. doi: 10.1128/AAC.02412-14 24777092 PMC4068535

[ref6] Coluzzi C. Pilar Garcillán-Barcia M. de la Cruz F. Rocha E. P. C. (2022). Evolution of plasmid mobility: origin and fate of conjugative and nonconjugative plasmids. Mol. Biol. Evol. 39:msac115. doi: 10.1093/molbev/msac115, PMID: 35639760 PMC9185392

[ref9005] De Coster W. D’Hert S. Schultz D. T. Cruts M. Van Broeckhoven C. (2018). “NanoPack: visualizing and processing long-read sequencing data.” Bioinformatics (Oxford, England) 34, 2666–2669. doi: 10.1093/bioinformatics/bty149 29547981 PMC6061794

[ref7] Evans D. R. Griffith M. P. Sundermann A. Shutt K. A. Saul M. I. Mustapha M. M.. (2020). Systematic detection of horizontal gene transfer across genera among multidrug-resistant bacteria in a single hospital. eLife 9:e53886. doi: 10.7554/eLife.53886 32285801 PMC7156236

[ref8] Finnish Institute for Health and Welfair. (2023). “CPE-Esiintyvyys Suomessa.” Available online at: https://thl.fi/fi/web/infektiotaudit-ja-rokotukset/taudit-ja-torjunta/taudit-ja-taudinaiheuttajat-a-o/salmonella/salmonellan-esiintyvyys-suomessa. (Accessed June 1, 2023).

[ref9] Hamprecht A. Sommer J. Willmann M. Brender C. Stelzer Y. Krause F.. (2019). Pathogenicity of clinical OXA-48 isolates and impact of the OXA-48 incL plasmid on virulence and bacterial fitness. Front. Microbiol. 10:2509. doi: 10.3389/fmicb.2019.02509 31736929 PMC6838017

[ref10] Katoh K. Standley D. M. (2013). MAFFT multiple sequence alignment software version 7: improvements in performance and usability. Mol. Biol. Evol. 30, 772–780. doi: 10.1093/molbev/mst010, PMID: 23329690 PMC3603318

[ref11] Kolho E. Lyytikäinen O. Jalava J. (2020). Ohje Moniresistenttien Mikrobien Tartunnantorjunnasta [National Guideline for Control of Multidrug-Resistant Microbes]. Helsinki: National Institute for Health and Welfare (THL).

[ref12] Kopotsa K. Osei Sekyere J. Mbelle N. M. (2019). Plasmid evolution in Carbapenemase-producing *Enterobacteriaceae*: a review. Ann. N. Y. Acad. Sci. 1457, 61–91. doi: 10.1111/nyas.14223, PMID: 31469443

[ref13] Li B. Feng J. Zhan Z. Yin Z. Jiang Q. Wei P.. (2018). Dissemination of KPC-2-encoding IncX6 plasmids among multiple Enterobacteriaceae species in a single Chinese hospital. Front. Microbiol. 9:478. doi: 10.3389/fmicb.2018.00478, PMID: 29616001 PMC5868456

[ref14] Marí-Almirall M. Ferrando N. Fernández M. J. Cosgaya C. Viñes J. Rubio E.. (2021). Clonal spread and intra- and inter-species plasmid dissemination associated with *Klebsiella Pneumoniae* Carbapenemase-producing Enterobacterales during a hospital outbreak in Barcelona, Spain. Front. Microbiol. 12:781127. doi: 10.3389/fmicb.2021.781127, PMID: 34867923 PMC8637019

[ref15] Marimuthu K. Venkatachalam I. Koh V. Harbarth S. Perencevich E. Cherng B. P. Z.. (2022). Whole genome sequencing reveals hidden transmission of Carbapenemase-producing Enterobacterales. Nat. Commun. 13:3052. doi: 10.1038/s41467-022-30637-5, PMID: 35650193 PMC9160272

[ref16] Orlek A. Stoesser N. Anjum M. F. Doumith M. Ellington M. J. Peto T.. (2017). Plasmid classification in an era of whole-genome sequencing: application in studies of antibiotic resistance epidemiology. Front. Microbiol. 8:182. doi: 10.3389/fmicb.2017.00182, PMID: 28232822 PMC5299020

[ref17] Poirel L. Bonnin R. A. Nordmann P. (2012). Genetic features of the widespread plasmid coding for the Carbapenemase OXA-48. Antimicrob. Agents Chemother. 56, 559–562. doi: 10.1128/AAC.05289-11, PMID: 22083465 PMC3256075

[ref18] Pournaras S. Protonotariou E. Voulgari E. Kristo I. Dimitroulia E. Vitti D.. (2009). Clonal spread of KPC-2 carbapenemase-producing *Klebsiella pneumoniae* strains in Greece. J. Antimicrob. Chemother. 64, 348–352. doi: 10.1093/jac/dkp207, PMID: 19525514

[ref19] Räisänen K. Lyytikäinen O. Kauranen J. Tarkka E. Forsblom-Helander B. Grönroos J. O.. (2020). Molecular epidemiology of Carbapenemase-producing Enterobacterales in Finland, 2012–2018. Eur. J. Clin. Microbiol. Infect. Dis. 39, 1651–1656. doi: 10.1007/s10096-020-03885-w, PMID: 32307627 PMC7427707

[ref20] Räisänen K. Sarvikivi E. Arifulla D. Pietikäinen R. Forsblom-Helander B. Tarkka E.. (2021). Three clusters of Carbapenemase-producing *Citrobacter Freundii* in Finland, 2016–20. J. Antimicrob. Chemother. 76, 2697–2701. doi: 10.1093/jac/dkab209, PMID: 34164687

[ref21] Redondo-Salvo S. Fernández-López R. Ruiz R. Vielva L. de Toro M. Rocha E. P. C.. (2020). Pathways for horizontal gene transfer in Bacteria revealed by a global map of their plasmids. Nat. Commun. 11:3602. doi: 10.1038/s41467-020-17278-2, PMID: 32681114 PMC7367871

[ref22] Robertson J. Nash J. H. E. (2018). MOB-suite: software tools for clustering, reconstruction and typing of plasmids from draft assemblies. Microb. Genom. 4:e000206. doi: 10.1099/mgen.0.000206 30052170 PMC6159552

[ref23] Schweizer C. Bischoff P. Bender J. Kola A. Gastmeier P. Hummel M.. (2019). Plasmid-mediated transmission of KPC-2 carbapenemase in Enterobacteriaceae in critically ill patients. Front. Microbiol. 10:276. doi: 10.3389/fmicb.2019.00276 30837980 PMC6390000

[ref24] Smillie C. Garcillán-Barcia M. P. Francia M. V. Rocha E. P. de la Cruz F. (2010). Mobility of plasmids. Microbiol. Mol. Biol. Rev. 74, 434–452. doi: 10.1128/MMBR.00020-10, PMID: 20805406 PMC2937521

[ref3] van Beek J. Räisänen K. Broas M. Kauranen J. Kähkölä A. Laine J.. (2019). Tracing local and regional clusters of carbapenemase-producing *Klebsiella pneumoniae* ST512 with whole genome sequencing, Finland, 2013 to 2018. Eur. Secur. 24:1800522. doi: 10.2807/1560-7917.ES.2019.24.38.1800522, PMID: 31552821 PMC6761573

[ref25] Van Duin D. Doi Y. (2017). The global epidemiology of Carbapenemase-producing Enterobacteriaceae. Virulence 8, 460–469. doi: 10.1080/21505594.2016.1222343, PMID: 27593176 PMC5477705

[ref9006] Wick R. R. Judd L. M. Gorrie C. L. Holt K. E. (2017). “Completing bacterial genome assemblies with multiplex MinION sequencing.” Microbial Genomics, 3. doi: 10.1099/mgen.0.000132 PMC5695209 29177090

[ref26] Zou X. Nguyen M. Overbeek J. Cao B. Davis J. J. (2022). Classification of bacterial plasmid and chromosome derived sequences using machine learning. PLoS One 17:e0279280. doi: 10.1371/journal.pone.0279280, PMID: 36525447 PMC9757591

